# Supplementary material for: Ubiquinol Ameliorates Endothelial Dysfunction in Subjects with Mild-to-Moderate Dyslipidemia: A Randomized Clinical Trial
Source: Nutrients. 2020 Apr 15;12(4):1098. doi: 10.3390/nu12041098 (PMC7231284; doi:10.3390/nu12041098)
Supplement: Supplementary file 1 [file nutrients-12-01098-s001.pdf]

Table S1. Inclusion and exclusion criteria as defined by the study protocol

**Inclusion criteria**
Males aged 35 - 65 years (35 and 65 included)
Post-menopausal females (without menses for > 1 year) aged up to 65 years (65 included)
Subjects recommended to adopt primary prevention but never treated (only lifestyle changes suggested as per current guidelines)
Screening FMD, 2.5-6.0%
Body mass index (BMI) from 18.5 to 29.9 kg/m^2
LDL cholesterol from 130 to 200 mg/dL, measured within 1 month prior to the screening visit
**Exclusion criteria**
Any drugs to treat cardiovascular diseases prior to the screening visit
Concomitant participation in any clinical trial
Change in body weight > 5% within one month of the screening visit
Participation in competitive physical sports
Lipid-lowering drugs or supplements – niacin (>100 mg/ day), garlic (> 600 mg/day), omega-3 fatty acids (> 1 g/day), red yeast rice extract, phytostanols / phytosterols (> 0.5 g/day), soluble fiber (>1 g/day), chitosan (> 1 g/day) and conjugated linoleic acid (> 3 g/day) – within 4 weeks prior to the screening visit
Phosphodiesterase inhibitors (e.g. sildenafil citrate, tadalafil, vardenafil) or nitric oxide donors, *e.g.* long-acting glyceryl trinitrate derivatives like. isosorbide dinitrate and amyl or butyl nitrite
Systemic corticosteroids (except nasal and inhaled corticosteroids), orlistat, bile acid resins, omega-3 fatty acids > 1 g, any hormone replacement therapy (estrogen or testosterone)
Consumption of > 2 alcohol units per day; 1 unit: 12 g ethanol, *e.g.* a small glass (125 ml) of medium gradation wine, a can of beer (330 ml) of average gradation or 40 ml of liquors

Flavonoid-enriched products
Vitamin C-enriched products or supplements containing vitamin C
A diagnosis of type 1 or type 2 diabetes mellitus, liver or kidney impairment or disease, thyroid disorders
Known cardiovascular disease or stroke, except for conditions that are deemed clinically insignificant by the Principal Investigator, Sub-investigator, or the study site physician (*e.g.* clinically insignificant atherosclerotic lesions observed by imaging studies)
History of significant gastrointestinal disease such as severe constipation, diarrhea, malabsorptive disease, inflammatory bowel disease (*e.g.* Crohn's disease, ulcerative colitis)
A history of severe psychiatric illness which in the opinion of the Principal Investigator would interfere with optimal participation in the study
A recent (< 5 years) history of cancer (except for successfully treated basal and squamous cell carcinoma of the skin)
Known HIV seropositivity
A history of bariatric surgery
Allergy to the product or the placebo
Smoking > 10 cigarettes/day
Risk of non-compliance with the study procedures or unsuitability for inclusion in the trial in the opinion of the Principal Investigator
